# Expected impact of MRI-targeted biopsy interreader variability among uropathologists on ProScreen prostate cancer screening trial: a pre-trial validation study

**DOI:** 10.1007/s00345-024-04898-2

**Published:** 2024-04-06

**Authors:** Ronja Hietikko, Tuomas Mirtti, Tuomas P. Kilpeläinen, Teemu Tolonen, Anne Räisänen-Sokolowski, Stig Nordling, Jill Hannus, Marita Laurila, Kimmo Taari, Teuvo L. J. Tammela, Reija Autio, Kari Natunen, Anssi Auvinen, Antti Rannikko

**Affiliations:** 1 Department of Urology, University of Helsinki and Helsinki University Hospital, Helsinki, Finland; 2 Research Program in Systems Oncology, Faculty of Medicine, University of Helsinki, Helsinki, Finland; 3 HUS Diagnostic Center, Department of Pathology, HUS Helsinki University Hospital, Helsinki, Finland; 4 Fimlab Laboratories, Department of Pathology, Tampere University Hospital, Tampere, Finland; 5 Department of Pathology, Helsinki University Hospital and University of Helsinki, Helsinki, Finland; 6 Department of Urology, Tampere University Hospital, Tampere, Finland; 7 Faculty of Social Sciences, Tampere University, Tampere, Finland

**Keywords:** Agreement, Interobserver, Grading, Kappa, Pathology

## Abstract

**Purpose:**

Prostate cancer (PCa) histology, particularly the Gleason score, is an independent prognostic predictor in PCa. Little is known about the inter-reader variability in grading of targeted prostate biopsy based on magnetic resonance imaging (MRI). The aim of this study was to assess inter-reader variability in Gleason grading of MRI-targeted biopsy among uropathologists and its potential impact on a population-based randomized PCa screening trial (ProScreen).

**Methods:**

From June 2014 to May 2018, 100 men with clinically suspected PCa were retrospectively selected. All men underwent prostate MRI and 86 underwent targeted prostate of the prostate. Six pathologists individually reviewed the pathology slides of the prostate biopsies. The five-tier ISUP (The International Society of Urological Pathology) grade grouping (GG) system was used. Fleiss’ weighted kappa (*κ*) and Model-based kappa for associations were computed to estimate the combined agreement between individual pathologists.

**Results:**

GG reporting of targeted prostate was highly consistent among the trial pathologists. Inter-reader agreement for cancer (GG1–5) vs. benign was excellent (Model-based kappa 0.90, Fleiss’ kappa *κ* = 0.90) and for clinically significant prostate cancer (csPCa) (GG2–5 vs. GG0 vs. GG1), it was good (Model-based kappa 0.70, Fleiss’ kappa *κ* 0.67).

**Conclusions:**

Inter-reader agreement in grading of MRI-targeted biopsy was good to excellent, while it was fair to moderate for MRI in the same cohort, as previously shown. Importantly, there was wide consensus by pathologists in assigning the contemporary GG on MRI-targeted biopsy suggesting high reproducibility of pathology reporting in the ProScreen trial.

## Introduction

Population-based prostate cancer (PCa) screening using prostate-specific antigen (PSA) and standard transrectal ultrasound-guided prostate biopsies in men with elevated PSA levels reduces cancer-specific mortality [1]. However, such screening also results in substantial overdiagnosis and overtreatment of clinically insignificant prostate cancer (cisPCa) [1, 2].

Multiparametric magnetic resonance imaging (mpMRI) of the prostate and subsequent targeted prostate biopsies of identified lesions with clinical suspicion of PCa (PI-RADS 3–5) are a promising diagnostic pathway [3]. In studies involving men with a suspected PCa, mpMRI improves the detection of csPCa and decreases cisPCa diagnosis compared to systematic biopsies [3]. A recent study by Eklund and colleagues showed that a pre-biopsy MRI only was not inferior to systematic biopsies for detecting csPCa (21% compared to 18%), while detection of cisPCa was reduced by two-thirds[4].

We initiated a population-based, prospective randomized PCa screening trial (ProScreen) in 2018. Unlike the STHLM3MRI and the Göteborg-2 studies, ProScreen trial is powered to evaluate PCa mortality as the primary endpoint [4, 5]. In the ProScreen trial, screen-positive men are referred to mpMRI with targeted prostate of the MRI visible lesion(s) only [6]. Thus, the emphasis is on minimizing overdiagnosis, while retaining the previously established PCa mortality reduction from screening. To this end, correct identification of csPCa by the pathologists is important for proper treatment selection. Further, to our best knowledge, no previous studies have been published on interobserver agreement of pathologists’ interpretation of MRI-targeted prostate biopsies. Importantly, the last ISUP consensus conference emphasized the differences between reporting of systematic biopsies and targeted prostate [7]. Therefore, the aim of this study was to evaluate MRI-targeted biopsy related interreader variability and its expected impact on the ProScreen trial.

## Materials and methods

We chose a cohort of 100 men who had been referred to the Helsinki University Hospital (HUS) for suspected PCa before the ProScreen trial. Men had varying baseline risk for PCa. The aim was to evaluate interreader variability in MRI and MRI-targeted biopsy. All 100 men were included in the previously reported study of interreader variability in MRI, and the cohort selection and patient demographics have been reported earlier [8]. For this study, 91 men had undergone MRI before diagnostic biopsies, whereas for 9 men, the MRI was used post-biopsy in cancer staging before definitive treatment. The biopsies were taken between June 2014 and May 2018 using MRI-fusion technique (UroNav, Philips, The Netherlands) to perform transrectal sampling of two to four biopsy cores per suspicious region of interest (ROI). Six patients’ samples could not be processed and viewed with cloud viewer due technical issues and were excluded from the final analysis.

All hematoxylin and eosin-stained glass slides of the 85 biopsies were included, representing the full spectrum of Gleason scores and no preselection of any kind was made. Slides were pseudonymized and digitally scanned using Pannoramic Flash III slide scanner (3D Histec, Budapest, Hungary) with a pixel resolution of 0.26 μm/pixel and reviewed with Aiforia cloud viewer software (Aiforia Technologies, Helsinki, Finland).

Six urological pathologists reviewed the slides and filled out a structured pathological assessment query including the number of glasses and biopsies, the length of biopsies and carcinoma, percent of Gleason pattern 4 or 5 and total ISUP Grade Group in each lesion. The pathology reports of the primary ROI (ROI1) were further analyzed. Clinical experience of the pathologists varied from 3 to 50 years (median 12.5, IQR 5.2–35.0). The pathologists were unaware of the other data regarding the patients. The observers were completely independent and were only given instructions to follow the current ISUP guidelines. No formal common training was organized. The original diagnostic pathology report on biopsies of ROI1 was collected.

### Statistical analysis

We analyzed the agreement between all pathologists using model-based kappa for association, which is the preferred method when there are more than two raters, and the classifications are ordinal [9]. Model-based kappa for association consider not only the exact agreement, but also the ratings close to each other, and the kappa value is computed giving weights to the classifications. Higher weights are given for the categories that are close to each other [10].

Along Model-based kappa, we have also used Fleiss kappa for comparison. Fleiss kappa is well known and more commonly used, but can be only used for categorical, not ordinal data. Fleiss kappa values and the Model based kappa for associations were computed with R using *irr* and *modelkappa* package. The interobserver agreement for grade groups are illustrated using R package *superheat*. Further the biopsies are clustered with k-means clustering into three groups, while the observers are clustered with hierarchical clustering using Euclidean distance and complete linkage. This retrospective analysis was evaluated and approved by the HUS Research Ethics Committee (HUS/333/2019).

## Results

**Fig. 1:**
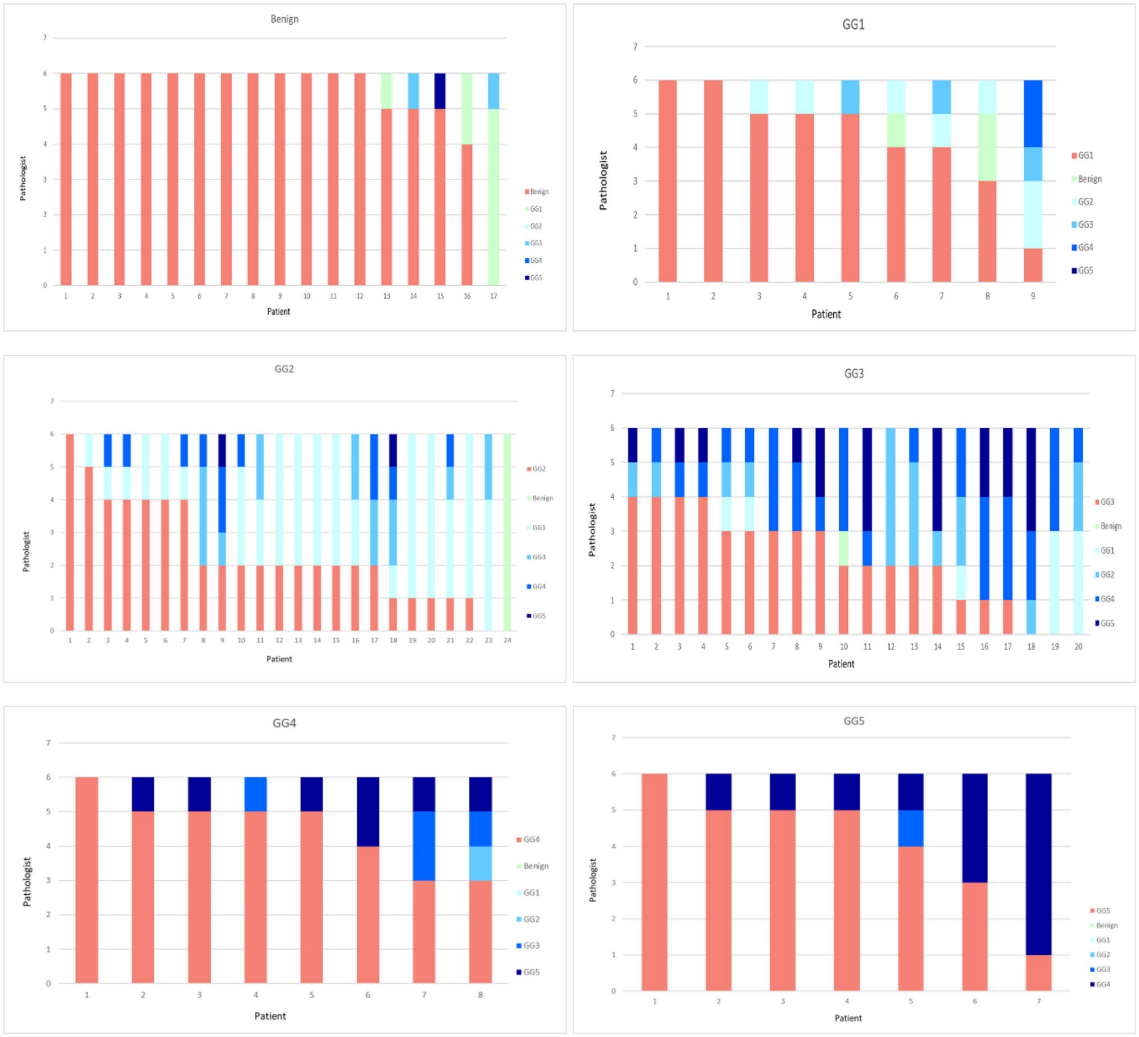
The number of pathologists identifying Gleason grade in prostate biopsies grouped by original pathological result

The median age of the study participants at biopsy was 68.8 years (interquartile range [IQR] 60.9–75.0) and the median PSA was 9.1 ng/ml (IQR 6.7–13.8). The median number of biopsies obtained from the index lesion was 2.5 (IQR 2.0–3.0). The reported median length of an individual biopsy was 11.9 mm (IQR 10.4–13.5) and the median length of the cancer in a particular biopsy was 4.9 mm (IQR 3.0–7.8) (Table 1). The distributions of the assigned grades for each case by the study pathologists are shown in Table 2. In the original diagnostic pathology reports, 69 men were diagnosed with PCa. The patient-level GG assessments of all the observers grouped by the original clinical pathology reports are illustrated in Fig. 1.

**Table 1:** Index lesion-wise comparison of pathological characteristics and agreement on aggregated GG between study pathologists

	Mean Bx length (mm)	Median	SD	Minimum	Maximum	Mean ca length (mm)	Median	SD	Minumum	Maximum	Pathologist experience (years)
Pathologist 1	11.9	11.8	2.5	4.5	23.0	5.9	5.0	3.5	0.5	17.0	15.0
Pathologist 2	12.7	12.5	2.5	6.0	24.0	6.3	6.0	3.6	0.3	17.0	10.0
Pathologist 3	12.1	12.0	2.9	5.0	24.0	5.0	4.5	3.3	0.5	15.0	50.0
Pathologist 4	12.7	12.4	2.6	1.3	23.7	6.1	5.7	3.3	0.4	17.1	3.0
Pathologist 5	12.0	11.9	2.7	5.7	23.7	6.3	5.4	5.7	0.1	17.3	6.0
Pathologist 6	11.0	10.9	2.9	1.7	23.2	5.5	5.1	3.4	0.2	17.2	30.0

	GG1–5 ca	GG3–5 ca
Agreement^1^ among pathologists 1–6	0.9	0.7

^1^Fleiss' kappa

**Table 2:** The distribution of the highest ISUP grade 1–5 defined for all patients from index lesion

Proposed grade	Clinical (%)	Pathologist 1 (%)	Pathologist 2 (%)	Pathologist 3 (%)	Pathologist 4 (%)	Pathologist 5 (%)	Pathologist 6 (%)	All pathologists (%)
GG0	20	22	20	20	19	20	18	20
GG1	11	16	22	35	4	31	21	22
GG2	29	15	17	6	20	16	20	16
GG3	21	19	12	8	18	14	11	14
GG4	11	24	15	26	22	11	11	18
GG5	8	4	14	5	18	8	20	11
GG1–5	80	78	80	80	81	80	82	80
GG2–5	69	61	58	45	78	49	61	59
GG3–5	40	46	41	39	58	33	41	43

We found complete agreement on the GG among all (6/6) pathologists in 18 of 85 (21.2%) cases. Of the 18 cases with complete agreement, 72.2% (13/18) were benign. We defined the consensus level as at least 2/3 agreement among the pathologists for a case according to the practice in all consensus meetings organized under the auspices of ISUP during the past decade [11]. The distribution of 2/3 grading consensus for ISUP GGs is shown in Table 3. With this criterion, consensus was reached for 65.9% (56/85) of the cases. The consensus grade differed from the initial grading in 13 cases. Almost all (92.3%) of these cases were in agreement within ±1 of the consensus GG.

**Table 3:** Reproducibility by proposed ISUP grade among all cases with the consensus level defined as at least 2/3 of all pathologist

ISUP GG	Consensus *n*/total *n*	Consensus for different GG, *n*	% consensus proposed grade	% consensus any grade
0	16/17	1^a^	94	100
1	7/10	0	70	70
2	7/25	9^b^	28	64
3	2/17	1^c^	12	18
4	6/9	1^d^	67	78
5	5/7	1^e^	71	86
0–5	56/85	−	−	66

^a^ An additional case reached consensus for ISUP grade 1

^b^ Eight additional cases reached consensus for ISUP grade 1, one case reached consensus for grade 0

^c^ An additional case reached consensus for ISUP grade 2

^d^ An additional case reached consensus for ISUP grade 3

^e^ An additional case reached consensus for ISUP grade 4

No consensus was reached (agreement among pathologists below 2/3) for 34.1% (29/85) of the cases. The most common source of disagreement was the estimated proportion of Gleason patterns 3 and 4. This reflects the challenges in distinguishing GG2 from GG3, as seen in six cases (21.4%).

**Fig. 2:**
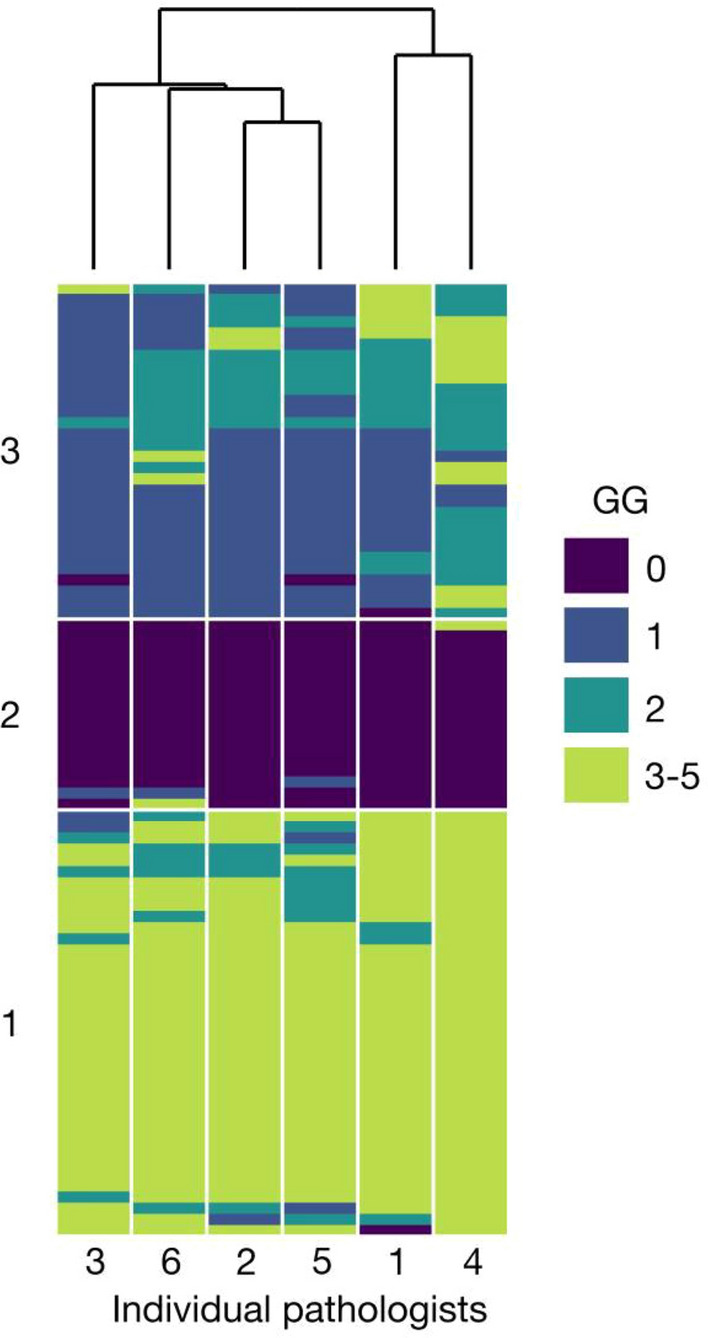
Heatmap visualization of the interobserver agreement for grade group. Individual pathologist are on the *x*-axis, colors represent grade groups for each ROI1 biopsy (GG 0 = benign). Pathologists and biopsies are ordered based on their similarity resulting from cluster analysis

The agreement among the observers in a comparison including all six categories of cancer and benign was good (Model-based kappa 0.65, 95% CI 0.59–0.70). Agreement among pathologists for cancer (GG1–5) vs. benign was excellent (Model-based kappa 0.90, Fleiss’ kappa *κ* = 0.90). For three-category comparison between csPCa (GG2–5) vs. cisPCa (GG1) vs. benign (GG0) the inter-observer agreement was good (Model-based kappa 0.70, Fleiss’ kappa 0.67). The heatmap visualization shows the interobserver agreement for grade groups (Fig. 2).

A total 23 patients had undergone radical prostatectomy until follow-up at the end of June 2019. Pathology report in 35% (*n* = 8) of these was disconcordant with the diagnostic pathology report of the MRI-targeted biopsies. Interestingly, and importantly, only 1 of 9 GG2 cancers at biopsy was upgraded to higher grade (one GG2 to GG3) at RP. This is important in the light of MRI-induced grade inflation and supports the notion that GG2 cancers perhaps should increasingly be offered active surveillance instead of immediate curative treatment [12]. However, we also found that the more biopsies were taken, the better concordance was achieved between the pathologist (Table 4).

**Table 4:** Inter-reader concordance correlated with mean number of biopsies in GG2–3 group

*n*/6 pathologists agree	Mean number of biopsies
6	4.0
5	3.3
4	2.8
3	3.0
1–2	2.5

## Discussion

Despite a recent recommendation by the European Commission to implement PCa in national screening programs, there is no high-quality scientific evidence from randomized screening trials confirming that MRI-based, or a novel biomarker-based, PCa screening would decrease mortality from prostate cancer.

Histological grading is one of the most important prognostic factors of PCa because of its validated prediction of the clinical behavior of cancer [9, 13]. Interestingly, previous screening trials have not assessed the diagnostic agreement of the pathological reporting prior to the study initiation [1, 14]. Nor have multi-center diagnostic trials comparing MRI-targeted biopsies to systematic biopsies [15, 16]. Here, we show that the interreader agreement among pathologists was good to excellent in grading of MRI-targeted biopsies. Therefore, the expected impact of variability on the MRI-based ProScreen screening trial is minimal. Sufficient agreement between pathologists is crucial for maintaining the value of the Gleason grading system as a diagnostic and prognostic tool and in determining the appropriate treatment for a patient [17]. According to some studies, 10–13% of PCa patients would receive different treatment recommendation after re-evaluation of biopsy specimen [15, 18, 19].

In the benign vs. cancer comparison, the more commonly used Fleiss’ kappa was similar to the model-based kappa (Fleiss’ kappa 0.90 vs. Model-based kappa 0.90). The model-based kappa is better suited for multi-categorical association analysis between several observers. Thus, it is not possible to directly compare our results with most of the previously published, systematic biopsy-based studies using Cohen’s or Fleiss’ kappa methodology. In addition, comparison across studies is challenging due to variation in definition of agreement, the type of investigated tissue (e.g., biopsies, radical prostatectomy specimens, transurethral resection specimens, a mixture of these, tissue microarray spots), different grouping of Gleason scores, the number of pathologists involved, and the number of specimens investigated. However, the agreement was better than in most other studies, in which kappa value has been calculated. The reported interobserver agreement among general pathologists for different comparisons has ranged from fair to moderate [20–22] although better results have also been reported [23]. The reproducibility among uropathologists tends to be better than among the general pathologists, usually ranging with between kappa values 0.56–0.67 [20, 23, 24]. We also noticed that the experience of the pathologist influences the results. Observer number four was an outlier in terms of years of experience. When we excluded this pathologist from the analysis, the agreement was higher among the more experienced pathologists.

Similar to our findings on MRI-related inter-reader variation [8], the extremes of the scale seem to be consistently reported, while the intermediate zone with borderline cases is challenging. We found the highest consensus with GG0 (100.0%) and GG5 (85.7%) and lower consensus within GG3 (25.0%) and GG2 (68.0%) cancer. In a PSA-based screening study, as many as 37.5% of biopsies were benign and only 8.0% of diagnosed cancers were GG3, suggesting that the overall reproducibility in a screening may be even better though MRI targeting likely has an impact on this [25]. The most common source of disagreement was separation of Gleason grade pattern 3 from pattern 4. The distinction between these two patterns was also recognized as a challenge in previous studies [23]. Egevad et al. found a specific challenge in differentiating tangentially cut GG1 from GG2 for cases with poorly formed or fused glands [26]. Further, fused glands or small glands without lumina may be interpreted as tangentially sectioned Gleason grade pattern 3 or as a focal Gleason grade pattern 4 [27]. Zhou et al. reported that any case with ≤5 poorly formed glands should not be graded as Gleason pattern 4 [28]. The ISUP 2014 revision of the Gleason grading system suggested that there should be more than occasional structures of this type for a tumor to qualify as Gleason pattern 4, otherwise they may represent tangential cuts. Previous studies have indicated that the reproducibility of Gleason pattern 4 with cribriform pattern is higher than Gleason pattern 4 with poorly formed or fused glands [11, 27]. All the above emphasize the importance of regular training, knowledge exchange between pathologists and intra-institutional peer evaluation, as making the decision on final GG is often subjective, especially in cases composed of Gleason grade patterns 3 and 4. The most evident difference in the fusion biopsy Gleason scoring was in the intermediate GG2/3 group. We suggest that these borderline GG2/3 biopsies should go through a second read. One practical solution for this could be an artificial intelligence-based model, which are available as commercial products and have been shown to improve decision making [29].

As the current clinical practice in diagnosing PCa relies heavily on targeted prostate alone or in conjunction with systematic biopsies, it is important that pathologists follow the guidelines for reporting [7]. Our study is the first to evaluate the interobserver agreement of multiple pathologists on MRI-targeted diagnostic prostate biopsies. Assessing a lesion-wise aggregate GG has been shown to correlate better with RP GG than core-wise highest GG [30], which again emphasizes the need to adhere to reporting guidelines.

The present study has some inherent limitations. MRI-targeted biopsies were obtained from men with clinical suspicion for PCa, not from a screening cohort. This may influence the generalizability of the study results to a screening study with lower underlying PCa risk. However, our study was not designed to assess the diagnostic performance, thus the related limitations such as high prevalence of the disease and verification bias are not essential. Further, the aim was to investigate the interreader agreement among the pathologist for targeted prostate biopsy specifically. Therefore, we chose a study cohort with relatively even distribution of different histopathologies. Moreover, contrary to clinical routine, pathologists were not allowed to consult a colleague when faced with challenging cases. This, however, likely underestimates the interreader agreement observed, especially in more aggressive cancers. When extrapolating the study results on the ProScreen trial, these limitations should not have a major effect as agreement between benign and cisPCa versus csPCa was good, thus supporting clinical decision making on cancer treatments. Given that the same teams of pathologists will evaluate PCa cases in both the screening and control arms, among screening participants and non-participants, any variability in grading is likely to results in nondifferential misclassification, and hence it is expected to slightly decrease the differences between the compared groups.

## Conclusion

The inter-reader agreement of MRI-targeted biopsy was good to excellent and better than the previously published inter-reader agreement for MRI from the same cohort. Therefore, it is plausible to assume that routine clinical histopathological evaluation is not likely to materially impact ProScreen trial results.

## Data Availability

The datasets used and analyzed during the current study are available from the corresponding author on reasonable request.
